# Virtual Namesake Point Multi-Source Point Cloud Data Fusion Based on FPFH Feature Difference

**DOI:** 10.3390/s21165441

**Published:** 2021-08-12

**Authors:** Li Zheng, Zhukun Li

**Affiliations:** School of Geodesy and Geomatics, Wuhan University, Wuhan 430079, China; lizhukun@whu.edu.cn

**Keywords:** iterative closest point, fast point feature histograms, virtual namesake point, point cloud registration

## Abstract

There are many sources of point cloud data, such as the point cloud model obtained after a bundle adjustment of aerial images, the point cloud acquired by scanning a vehicle-borne light detection and ranging (LiDAR), the point cloud acquired by terrestrial laser scanning, etc. Different sensors use different processing methods. They have their own advantages and disadvantages in terms of accuracy, range and point cloud magnitude. Point cloud fusion can combine the advantages of each point cloud to generate a point cloud with higher accuracy. Following the classic Iterative Closest Point (ICP), a virtual namesake point multi-source point cloud data fusion based on Fast Point Feature Histograms (FPFH) feature difference is proposed. For the multi-source point cloud with noise, different sampling resolution and local distortion, it can acquire better registration effect and improve the accuracy of low precision point cloud. To find the corresponding point pairs in the ICP algorithm, we use the FPFH feature difference, which can combine surrounding neighborhood information and have strong robustness to noise, to generate virtual points with the same name to obtain corresponding point pairs for registration. Specifically, through the establishment of voxels, according to the F2 distance of the FPFH of the target point cloud and the source point cloud, the convolutional neural network is used to output a virtual and more realistic and theoretical corresponding point to achieve multi-source Point cloud data registration. Compared with the ICP algorithm for finding corresponding points in existing points, this method is more reasonable and more accurate, and can accurately correct low-precision point cloud in detail. The experimental results show that the accuracy of our method and the best algorithm is equivalent under the clean point cloud and point cloud of different resolutions. In the case of noise and distortion in the point cloud, our method is better than other algorithms. For low-precision point cloud, it can better match the target point cloud in detail, with better stability and robustness.

## 1. Introduction

In recent years, point cloud data have been applied to more field, such as robots and autonomous driving, face recognition, gesture recognition, etc. In the face of autonomous driving systems above L3, high-precision maps have become an indispensable part. A high-precision map is a special map with centimeter-level accuracy and detailed lane information compared to general navigation maps. It can describe road more comprehensively and in detail and reflect the real situation of the road more accurately [1]. High-precision maps are one of the important applications of high-precision point cloud. There are three methods of obtaining high-precision map point cloud data: mobile surveying vehicle collection, drone aerial survey and 1:500 topographic map [2]. The sensors utilized in each data acquisition scheme are different due to the heterogeneity of sensors. The point cloud data obtained are thus very different in accuracy and range from the data set. Specifically, some sensors obtain point cloud with high accuracy but small range, and other sensors obtain point cloud with low accuracy but large range, etc. Therefore, how to fuse the point cloud data obtained by different sensors and combine their respective advantages is the key to generating high-precision maps. In the development of three-dimensional city model reconstruction, it has undergone changes from a single data source to multi-source data integration. There have been some studies using oblique photography, vehicle-borne light detection and ranging (LiDAR) or multi-source point cloud data fusion to perform 3D reconstruction [3,4].

Point cloud registration is a very important part of the point cloud fusion process. At present, the existing research in this area mostly is implemented by reference point cloud precision registration. There are more mature algorithms in point cloud registration. It is iterative closest point (ICP) proposed by Besl and Mckay et al., and normal distributions transform proposed by Biber et al. [5,6]. The ICP algorithm is to find corresponding points in two existing point cloud data. Due to various factors such as sensors and scanning angles, these corresponding points cannot be true corresponding points. The registration result will have a certain error; at the same time, the ICP algorithm can only have a rigid transformation on target point cloud as a whole but cannot correct all points in the target point cloud. This is also the shortcoming of ICP algorithm in multi-source point cloud registration.

Deep learning allows the machine to learn from human activities to achieve the effect of imitation, in order to solve some complex problems [7]. Some scholars have conducted research on point cloud registration combined with deep learning technology. Elbaz G, Avraham T and others used convolutional neural networks to collect local features and completed point cloud registration [8]. In 2019, Baidu unmanned vehicles proposed the first end-to-end high-precision point cloud registration network [9].

Our main work is as follows.

- By introducing Fast Point Feature Histograms (FPFH) features, using the CNN network to learn the F2 distance of FPFH features to obtain the probability, improve the robustness to point cloud noise and point cloud resolution.
- Use voxels that use spatial information around the point to generate virtual points, increasing the accuracy and stability of finding corresponding points.
- Show the results compared to other existing works on experimental evaluations under clean, noisy, different resolution and distorted datasets.

The remainder of the paper is organized as follows: We begin with a review of related work in Section 2. The main steps of our method are described in detail in Section 3. Our experiment results are discussed in Section 4, and conclusions are drawn in Section 5.

## 2. Related Work

Multi-source point cloud fusion is actually the precise registration of two point clouds with differences in accuracy, resolution and range obtained from the same scene scan. ICP is a commonly used algorithm for precision registration, it is an optimal matching algorithm based on the least squares. The algorithm uses the closest point as the corresponding point in the target point cloud and the source point cloud, and takes the distance between the closest point pair as the target, calculates the optimal rigid body transformation between the point cloud to complete the point cloud matching

In 1992, Besl and Mckay et al. proposed the nearest point iterative algorithm to realize free-form surface registration and automatic registration of original point cloud, which became the basic algorithm for automatic point cloud registration [5]. ICP algorithm selects the point with the smallest Euclidean distance as the corresponding point, calculates the rigid transformation matrix with corresponding point pairs and iteratively obtains the optimal transformation. Using the shortest Euclidean distance as the judgment standard to determine the corresponding point directly can find the corresponding point simply and quickly, but it will cause a lot of mismatches and reduce the registration accuracy. At the same time, the ICP algorithm may easily become trapped in a local minimum when there is no good initial position or when there is noise in the point cloud. Aiming at the goal of improving the correct rate of corresponding points, scholars have proposed a series of improvements. Censi et al. proposed using the distance from the point to the line to determine the corresponding point. Even though this method avoids the shortest distance search and improves the search efficiency, the registration accuracy is reduced [10]. Chen et al. added the point cloud normal vector to the original ICP algorithm and improved the point-to-point model to the point-to-surface model [11]. On the basis of Chen, Mitra et al. calculated the distance between the corresponding points of the two point clouds based on the idea of geometric Euclidean distance, and used different methods for registration by using the distance [12]. Luo et al. formed the three points closest to target point cloud to form a triangle and took the perpendicular foot of the triangle as the corresponding point [13]. Segal et al. proposed the G-ICP algorithm, which uses the covariance matrix to play a role similar to weights, eliminates some bad corresponding point pairs and creatively integrates point-to-point ICP and point-to-surface ICP [14].

With the increasing development of technology, deep learning, a subfield of machine learning, has been applied in various fields. In point cloud registration, deep learning is also involved. Elbaz et al. [8] used convolutional neural networks to gather local features to complete point cloud registration. Li Danyu [15] used the convolutional neural network structure of three orthogonal views to distinguish the target and other objects in the 3D point cloud candidate by filtering and matching the data set. In 2019, Baidu unmanned vehicles proposed the first end-to-end high-precision point cloud registration network that achieves comparable registration accuracy to prior state-of-the-art geometric methods [9]. The basic idea is to match dozens of robust key points which are extracted by the module named Weighting Layer in the target point cloud and the source point cloud. First introduce semantic features to automatically avoid dynamic features and select key points that are easy to match, generate grid points on key points, regenerate features for key points and grid points and use the feature difference between key points and grid points to calculate a matching probability, fusing the probability to generate a virtual and robust point with the same name for optimized pose calculation.

These improved methods have a prerequisite, that is, it is assumed that the corresponding point pairs found in the target point cloud and the source point cloud are exactly the same. However, due to different sensors, different scanning angles, different scanning resolutions and other factors, it is difficult to guarantee that the coordinates of the corresponding point pairs representing a certain space in reality are exactly the same. These corresponding point pairs have a certain coordinate error. In the literature [9], the concept of virtual points with the same name is proposed, but the literature [9] mainly acts on the point cloud data obtained by two pieces of the same sensor and focuses on excluding the selection of key points on dynamic objects, which is not suitable for different sensors. For the registration of point cloud data with different accuracy, this article proposes virtual namesake point multi-source point cloud data fusion based on FPFH feature difference. This method does not intend to find the corresponding point with the same name in the existing points of the target point cloud, but directly generates a virtual corresponding point with the same name based on the method of deep learning, which improves the accuracy of the ICP algorithm. At the same time, it is used for overlapping areas and for low-precision points. The cloud has been corrected to a certain extent to make it more closely fit the high-precision point cloud.

## 3. Methodology

**Figure 1:**
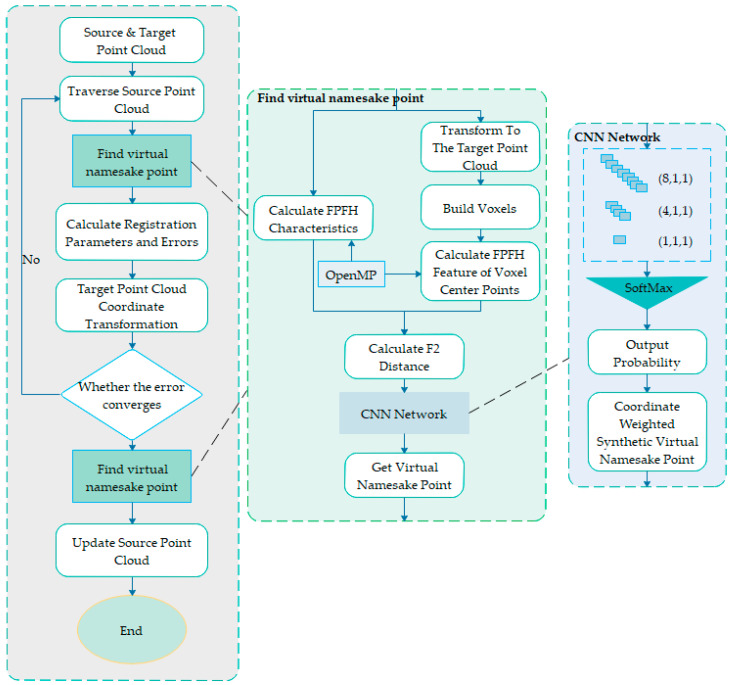
Flow chart of virtual namesake point multi-source point cloud data fusion based on FPFH feature difference.

Aiming at the problem of multi-source point cloud data registration with different accuracy, different resolution and noise, this paper proposes a virtual namesake point multi-source point cloud data fusion based on FPFH feature difference to improve the traditional ICP algorithm. In the ICP algorithm, the step of finding the corresponding point is replaced with a virtual point search based on the FPFH feature difference. In this process, a K-dimensional tree [16] search is used. The K-dimensional tree is a data structure that divides the K-dimensional data space. It is used to represent a collection of points in K-dimensional space. The traditional ICP algorithm consumes a lot of time in the process of repeated iterative search for the closest point, which reduces the computational efficiency. Therefore, using the K-dimensional tree to search for the corresponding point can effectively reduce the computational complexity and realize the fast search of the neighborhood relationship. Embed the multi-core and multi-threaded OpenMP parallel processing mode to accelerate the fast feature histogram extraction of key points while extracting FPFH features. After performing the optimal rigid transformation, the low-precision target point cloud is corrected in detail to obtain a better-precision corresponding points and conversion matrix. Therefore, the low-precision point cloud is better match the high-precision point cloud. The flowchart of this method is shown in Figure 1.

Our method mainly includes the following key processes:

(1) Finding virtual namesake points based on FPFH feature difference: calculate the FPFH eigenvector value $FPFH_{p_{i}}$ of the current point $p_{i}(X_{i},Y_{i},Z_{i})\left\{p_{i}\in R^{3},i=1,\ldots ,N_{p}\right\}$ in the source point cloud P, and then convert the current point $p_{i}(X_{i},Y_{i},Z_{i})$ to the target point cloud to acquire the point $p_{i}^{\prime }(X_{i}^{\prime },Y_{i}^{\prime },Z_{i}^{\prime })$, and construct a 2*2*2 voxel around the point $p_{i}^{\prime }(X_{i}^{\prime },Y_{i}^{\prime },Z_{i}^{\prime })$ to acquire 8 voxel center points, the center point of each voxel is set to point $q_{j},j=1,\ldots ,8$ the above 8 voxel center points are, respectively, interpolated into the target point cloud Q, and the FPFH value of the voxel center point $q_{j},j=1,\ldots ,8$ is calculated after interpolation. This will obtain the feature vector value of $FPFH_{q_{j}},j=1,\ldots ,8$ calculate the F2 distance of $FPFH_{q_{j}},j=1,\ldots ,8$ and $FPFH_{p_{i},}$ respectively, will acquire 8 F2 distances and input them into the constructed CNN convolutional neural network, and output the probability $w_{j},j=1,\ldots ,8$ corresponding to the center point $q_{j}$ of each voxel, thus according to the coordinate $q_{j}$ of the prime center point and the corresponding probability $w_{j}$ form a virtual point $q_{i}^{\prime }$, and the virtual point $q_{i}^{\prime }$ is selected as the corresponding point of the current point in the source point cloud.
(2) Point cloud registration algorithm based on virtual points with the same name: based on the ICP algorithm, replace the step of using the closest point as the point with the same name in the ICP algorithm to find the virtual point with the same name in (1), and perform the target point cloud and the source point cloud Point cloud registration.
(3) Further correction of overlapping area point cloud: After the overall best point cloud registration of the low-precision point cloud, further precision corrections can be made to the details. Once again, we acquire virtual namesake points as we did in step 1, then replace the low precision points with these virtual namesake points. Correct the accuracy of the points in the low-precision point cloud to improve the accuracy of the low-precision point cloud.

### 3.1. Finding Virtual Namesake Point Based on FPFH Feature Difference

The process of finding virtual namesake point in this paper is divided into two steps. The first step is to extract the F2 difference of FPFH features, and the second step is to calculate the coordinates of the virtual namesake point.

**Figure 2:**
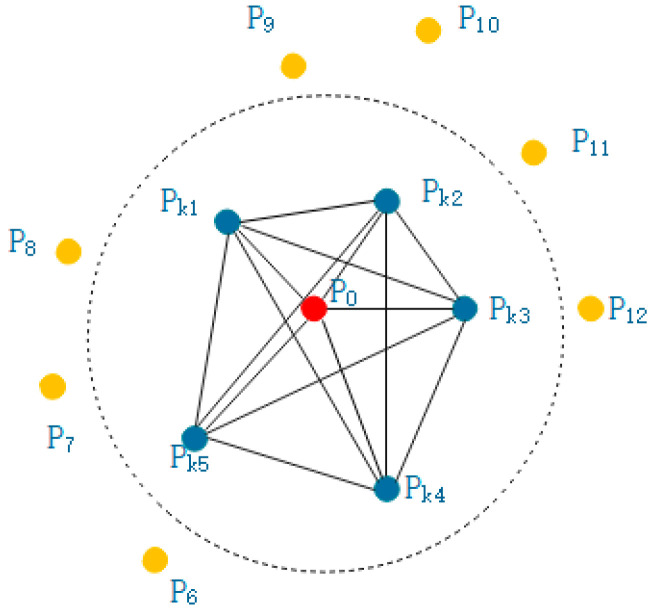
Schematic diagram of Point Feature Histogram neighborhood.

**Figure 3:**
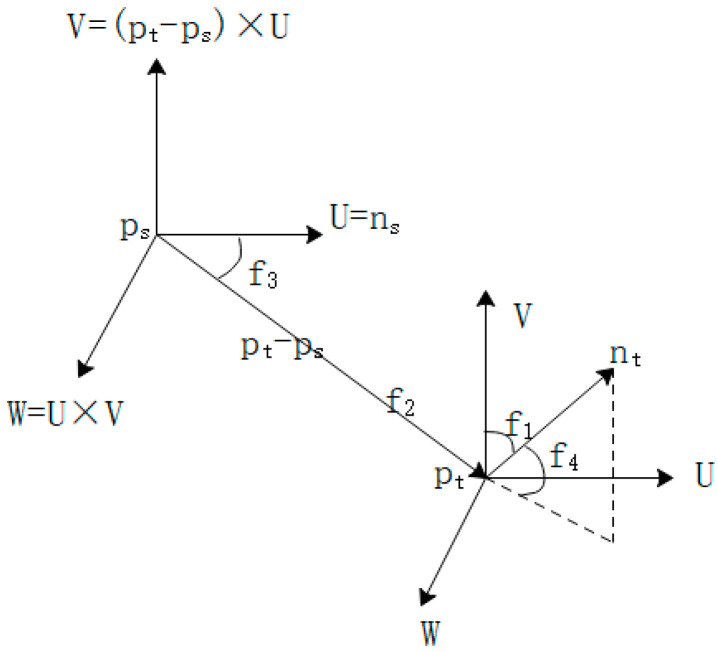
Local coordinate system between point pairs.

In the first step of extracting the F2 difference of FPFH features, we select FPFH [17]. There are two reasons for the selection. The first reason is the advantages of FPFH’s own features. Since the histogram is in a high-dimensional hyperspace, it provides a measurable information space for the feature representation of the point cloud, and FPFH has good robustness to point cloud noise. It can still work under different scanning resolutions or scanning accuracy. The corresponding point can be found correctly. The second reason is that the FPFH feature is a feature descriptor that combines the information of the surrounding neighborhood points. It is necessary to refer to the difference between the conversion point and the surrounding neighborhood while calculating the virtual namesake point. FPFH can provide a high dimensional vector and make full use of neighborhood spatial information to improve accuracy. Here is a brief introduction to the steps of FPFH feature extraction:

(1) Suppose the point cloud P is known and its coordinates and r neighborhood are known, that is, a sphere is made with point $p_{0}$ as the center and r as the radius. The points surrounded by the sphere are the neighborhood of point $p_{0}$, as shown in Figure 2. As shown, $P_{k1},P_{k2},P_{k3},P_{k4},P_{k5}$ are neighborhood points, and each point in the neighborhood is connected in pairs, forming a point pair with each other.
(2) Construct the local coordinate system of the point pair as shown in Figure 3:
  (1) $$\{\begin{array}{c} u=n_{s}\\ v=\left(p_{t}-p_{s}\right)\times u\\ w=u\times v \end{array}$$
  where u,v,w is the coordinate axis of the coordinate system.
(3) At this time, according to the normal vector and coordinate system of the point pair, calculate the four eigenvalues $f_{1},f_{2},f_{3},f_{4}$ of the point pair:
  (2) $$\{\begin{array}{c} f_{1}=\langle v,n_{t}\rangle \\ f_{2}=\|p_{t}-p_{s}\|\\ \begin{array}{c} f_{3}=\langle u,p_{t}-p_{s}\rangle /f_{2}\\ \;f_{4}=\tan^{-1}\left(\langle w,n_{t}\rangle ,\langle u,n_{t}\rangle \right) \end{array} \end{array}$$

After calculating the four eigenvalues of each point pair in the neighborhood, each eigenvalue is divided into five intervals. At this time, since there are three eigenvalues, there are $5^{3}=125$ intervals, so a 125-dimensional histogram will be generated. It is a 125-dimensional feature Point Feature Histogram (PFH).

**Figure 4:**
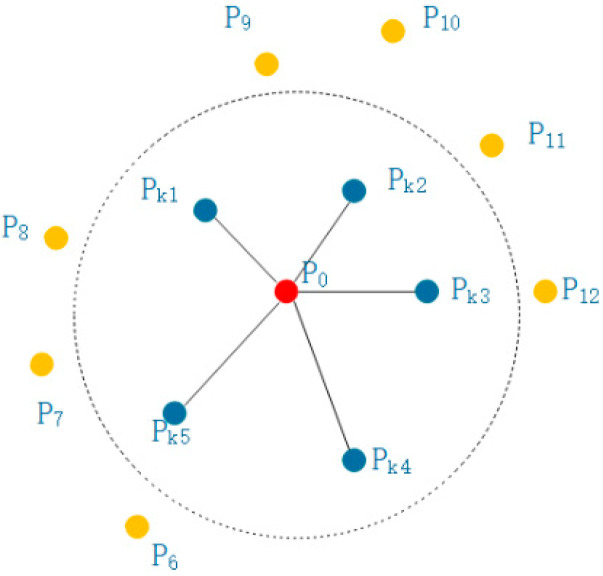
Schematic diagram of FPFH constructing r neighborhood.

The FPFH feature is the feature obtained through the integration of simplified PFH [18] features. The neighborhood construction is shown in Figure 4, which only connects the center point and the neighboring points compared to Figure 2. In the above eigenvalues, only select $f_{1},f_{3},f_{4}$, and divide each eigenvalue into 11 intervals according to the range, so that there are 33 eigenvalues representing SPFH features and then weight the SPFH features in the neighborhood to acquire FPFH feature.

(3) $$\mathrm{FPFH}=\mathrm{SPFH}+\frac{1}{k}\sum_{i=1}^{k}\frac{1}{w_{k}}\cdot SPFH\left(p_{k}\right)$$

where $w_{k}$ is the weight, which represents the Euclidean distance between the point pairs.

The second step is to calculate the coordinates of the virtual namesake point. Deep learning is implemented by Convolutional Neural Networks (CNN) and SoftMax. For the input part of the CNN, the first step is to calculate it for a point $p_{i}(X_{i},Y_{i},Z_{i})\left\{p_{i}\in R^{3},i=1,\ldots ,N_{p}\right\}$ in the source point cloud P, the FPFH feature $FPFH_{p_{i}}$ of, and keep it. Then use the initial transformation matrix R,T to perform coordinate transformation on the point $p_{i}$, and call the transformed point $p_{i}^{\prime }(X_{i}^{\prime },Y_{i}^{\prime },Z_{i}^{\prime })$ the transformation point. Put the transformation point $p_{i}^{\prime }$ into the target point cloud. Through the neighborhood search method, the neighborhood is divided into $\left(\frac{2r}{s}+1,\frac{2r}{s}+1,\frac{2r}{s}+1\right)$ voxels, where r is the width of the neighborhood, s is the size of the voxel, and each voxel in it contains some points in the target point cloud.

**Figure 5:**
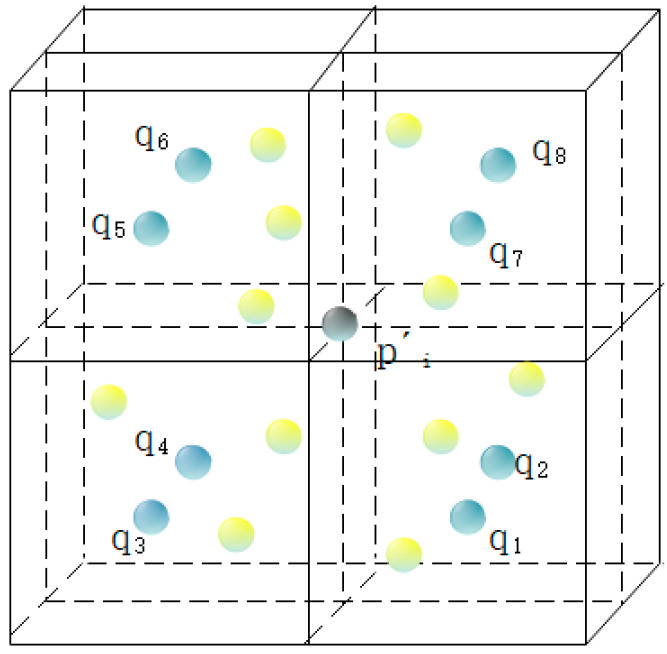
Construct voxel diagram.

**Figure 6:**
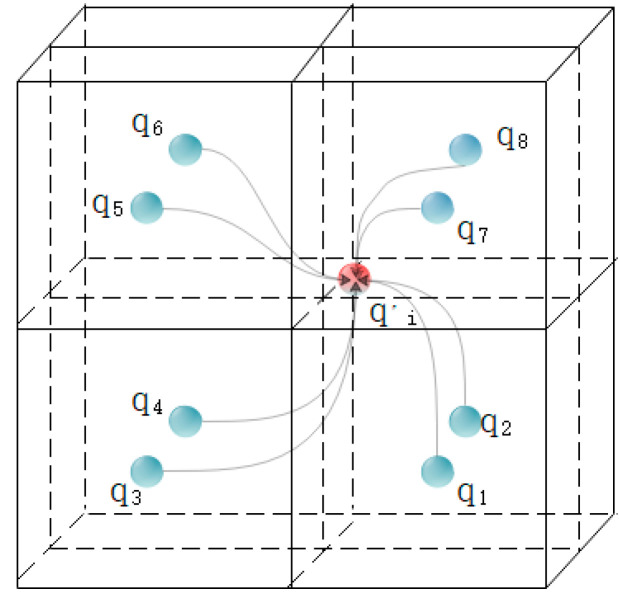
Virtual namesake point synthesis.

As shown in Figure 5, there are 8 voxels after the current point is divided into voxels, where $q_{j},j=1,\ldots ,8$ is the center point of the voxel, $p_{i}^{\prime }$ is the point converted to the target point cloud, and the other points are the neighbors of the conversion point in the target point cloud. Domain point. Then in each voxel, use other points in the voxel to extract the FPFH feature of the voxel center point $q_{j}$, and at this time, the FPFH feature $FPFH_{q_{j}},j=1,\ldots ,8$ of the 8 voxel center points will be obtained. Finally, calculate the F2 distance between the FPFH feature $FPFH_{q_{j}},j=1,\ldots ,8$ of the 8 voxel center points and the FPFH feature $FPFH_{p_{j}}$ of the point $p_{i}$ in the source point cloud, respectively, which is used as the input of 3D CNNS, and the softmax operation can complete the whole step. CNN can learn the similarity distance measurement between the source feature and the target feature. More importantly, it can suppress the matching noise. The SoftMax operation is used to convert the matching cost into a probability, denoted by $w_{j},j=1,\ldots 8$. The finally generated virtual namesake point are calculated by Formula (4), as shown in Figure 6:

(4) $$q_{i}^{\prime }=\frac{1}{\sum_{j=1}^{8}w_{j}}\sum_{j=1}^{8}w_{j}q_{j}$$

Through CNN, you can use its powerful expression ability in similarity learning, can automatically learn deeper and more abstract feature information and directly find the corresponding virtual namesake point, avoiding the operation of finding the corresponding point in the existing point. Improve the accuracy of finding corresponding points, thereby improving the accuracy of point cloud matching.

### 3.2. Point Cloud Registration Algorithm Based on Virtual Namesake Point

The entire improved point cloud registration algorithm process is similar to the classic ICP algorithm. The biggest change is to use the virtual point search method proposed in Section 3.1. to replace the closest point in the classic ICP algorithm as the corresponding point process. The algorithm is summarized as follows:

(1) First select two point cloud datasets P,Q with different accuracy, take the low-precision point cloud P as the source point cloud, the relatively high-precision point cloud Q as the target point cloud and the points $p_{i},p_{i}\in R^{3},i=1,\ldots ,N_{p}$ in the source point cloud as the candidate points.
(2) Use the initial conversion matrix R,T to transform all points $p_{i}$ in the source point cloud and convert all points $p_{i}$ in the source point cloud to obtain the conversion points $p_{i}^{\prime },p_{i}^{\prime }\in R^{3},i=1,\ldots ,N_{p}$.
(3) Put all the obtained conversion points $p_{i}^{\prime }$ into the target point cloud Q, find the neighbor points of the conversion point in the target point cloud Q, set a threshold r at this time, calculate the Euclidean distance d between the conversion point and the nearest neighbor point in the target point cloud, compare the calculated distance with the threshold. If it is greater than the threshold, it indicates that the conversion point does not overlap in the source point cloud, delete the conversion point and keep the conversion point $q_{i},q_{i}\in R^{3},i=1,\ldots ,N_{q}$ less than the threshold.
(4) Perform FPFH eigenvalue calculation on the conversion point $q_{i}$ obtained above in the source point cloud to obtain $FPFH_{p_{i}}$.
(5) Find the neighborhood of the conversion point $q_{i}$ in the target point cloud and divide the voxel to obtain the voxel center point $q_{i},j=1,\ldots ,8$.
(6) Calculate the FPFH eigenvalue $FPFH_{q_{i}},j=1,\ldots ,8$ of the voxel center point $q_{i},j=1,\ldots ,8$ and calculate the F2 distance from $FPFH_{p_{i}}$.
(7) Feed the F2 distance obtained in (6) into the CNN network to obtain the probability $w_{j},j=1,\ldots ,8$ of the voxel center point.
(8) Use the probability $w_{j},j=1,\ldots ,8$ in (7) to calculate the virtual point $q_{i}^{\prime }$ corresponding to the conversion point $q_{i}$.
  (5) $$q_{i}^{\prime }=\frac{1}{\sum_{j=1}^{8}w_{j}}\sum_{j=1}^{8}w_{j}q_{j}$$
(9) After obtaining the corresponding points, calculate the conversion matrix R′,T′ according to the least squares, and the calculation principle is to minimize the objective function of Formula (6):
  (6) $$e\left(R,T\right)=\frac{1}{w}\sum_{N_{p}}\omega_{k}\|R^{\prime }q_{i}+T^{\prime }-{q_{i}}^{2}\|{}^{2}$$
(10) Repeat the above steps until the number of cycles is reached or the objective function is basically unchanged.

### 3.3. Point Cloud in Overlapping Areas for Further Correction

In the previous section, the low-precision point cloud was registered with reference to the high-precision point cloud, and the optimal rigid transformation matrix was calculated. However, this is only a rotation and translation operation for the entire point cloud. The geometric difference of the point cloud may be different, so the improvement of the point cloud accuracy of the ICP algorithm is limited to the whole, which requires further improvement in the detailed area.

**Figure 7:**
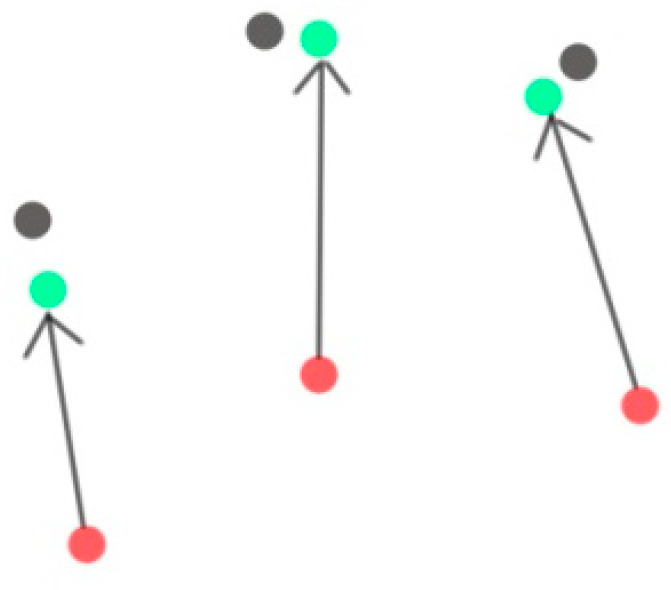
Position correction: the black point in Figure 7 is the high-precision point cloud, the red point is the point cloud to be registered and the green is the point cloud after registration.

For the point cloud in the overlapping area of the source point cloud and the target point cloud, after performing the point cloud registration algorithm operation based on the virtual namesake point, it is necessary to search for the virtual namesake point based on the FPFH feature difference again, that is, this time in the existing Under the optimal initial position, find the virtual namesake point in the low-precision point cloud for improvement. The improvement steps are similar to those in Section 3.2, and the operations in Section 3.2 are performed on all candidate points marked in Section 3.3. As shown in Figure 7, the black point in Figure 7 is the high-precision point cloud, the red point is the point cloud to be registered and the green is the point cloud after registration. In detail, the correction direction and size of each point can be based on the characteristics of the point itself. Correction, this approach can not only achieve fine fitting in a small range, but also keep the correction amount between regions without large jumps as a whole and ensure the continuity of the entire map area.

## 4. Experiment and Result Discussion

### 4.1. Experimental Data and Baseline Algorithms

The experimental data in this paper come from the data set WHU-TLS dataset released by the research group of the Institute of Space Intelligence of Wuhan University and the 3D scanning repository of Stanford [19,20,21]. The WHU-TLS dataset contains a total of more than 1.74 billion 3D points collected from 11 different environments of the ground station scanning point cloud data set. We select some representative four types of point cloud data for experimentation, including Bunny, Sign Board, Sculpture and Chair, which correspond to the data in the result graph below. There are different point cloud resolutions, local distortions and noise between multi-source point clouds. In order to verify the ability of our method to deal with the differences between multi-source point clouds, we performed Gaussian noise addition, point cloud downsampling and point cloud distortion processing on the experimental data, respectively. We compare the performance of ours with the following registration algorithms: ICP [5], Normal Distributions Transform (NDT) [6] and Fast Global Registration (FGR) [22].

### 4.2. Evaluation Metrics

We evaluate the registration by computing the mean isotropic rotation and translation errors:

(7) $$R_{id}=R_{i}R_{GT}^{-1}$$

(8) $$\mathrm{Error}\left(R\right)=\cos^{-1}\left(\frac{trace\left(R_{id}\right)-1}{2}\right)\frac{180}{\pi }$$

(9) $$\mathrm{Error}\left(t_{id}\right)=\|t_{GT}-t_{i}\|{}_{2}$$

where $R_{id}$, $t_{id}$ is the real rotation matrix and translation vector, and $R_{i},t_{i}$ is the rotation matrix and translation vector calculated by the algorithm. The error Error(R) of the rotation matrix is calculated by Formula (8) and expressed by angle. Similarly, the translation error $\mathrm{Error}\left(t_{id}\right)$ is expressed by Formula (9).

In addition, the Chamfer Distance (CD) is used to evaluate the distance between the point clouds. If there are two point clouds $S_{1},S_{2}$, the Chamfer Distance (CD) is calculated by Formula (10).

(10) $$D\left(S_{1},S_{2}\right)=\frac{1}{S_{1}}\underset{x\in S_{1}}{\sum }min_{y\in S_{2}}\|x-y\|{}_{2}^{2}+\frac{1}{S_{2}}\underset{y\in S_{2}}{\sum }min_{x\in S_{1}}\|x-y\|{}_{2}^{2}$$

### 4.3. Experiment Analysis

**Figure 8:**
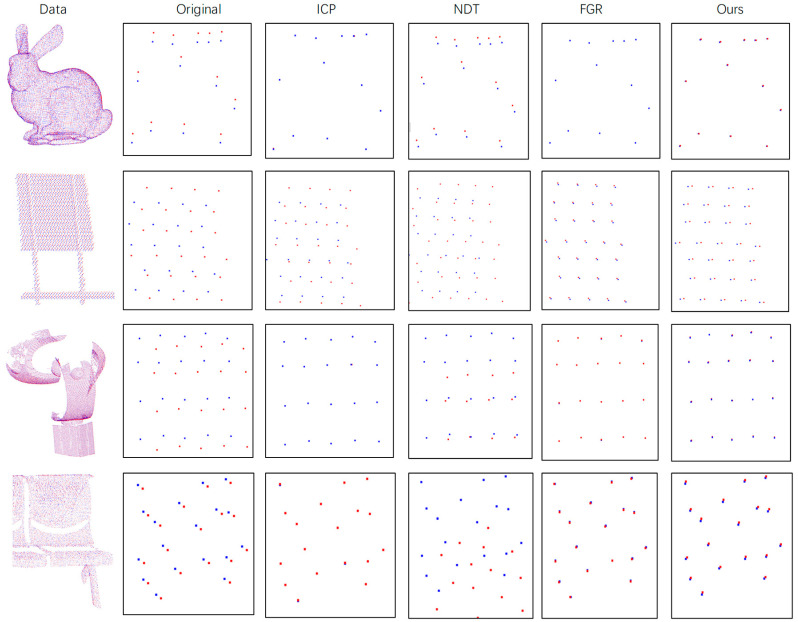
Performance on Clean Data. The first column on the left of the figure is the original point cloud data, the second column is the detailed schematic diagram and the rest are the detailed diagrams after registration by each method.

**Clean Data:** We do not perform any processing on the four types of point cloud data and apply a random rigid transformation matrix while keeping it in a clean state. Each type of point cloud data has a correct corresponding relationship for the accuracy evaluation of the algorithm. The experimental results are shown in Table 1. It can be seen that in the clean data, the ICP algorithm is the most accurate overall, but if the data Sign Board falls into the local optimum, FGR is the most stable. Ours is slightly worse than FGR, but FGR is very noise-sensitive. Sensitive, overall, ours is better than NDT. The qualitative results are shown in Figure 8. The red point cloud represents the transformed source point cloud, the blue point cloud represents the target point cloud, because this method is mainly for the precise registration of the test. The effect is shown here with the enlarged image in the data.

**Figure 9:**
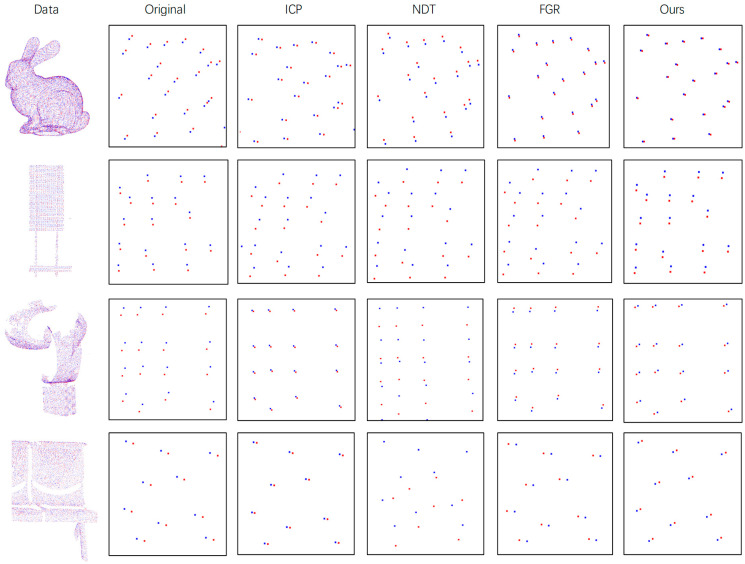
Performance on Different Resolution Data. The first column on the left of the figure is the original point cloud data, the second column is the detailed schematic diagram and the rest are the detailed diagrams after registration by each method.

**Different Resolution Data:** The resolution of point cloud data from different sources is not necessarily the same but can be dense or sparse. In order to simulate the difference in point cloud sampling in reality, we down-sampled the experimental data to test the registration effect at different resolutions. The simulated data are roughly down-sampled to two-thirds of the original points. Experimental results are shown in Table 2. It can be seen that in point cloud data with different resolutions, our algorithm is superior to other methods, except that it is occasionally lower than ICP, but in most cases the ICP falls into the local optimum. The qualitative results are shown in Figure 9. The red point cloud represents the transformed source point cloud, the blue point cloud represents the target point cloud. For better display effects, the details are enlarged. The target point cloud in the figure is also down-sampled but note that the target point cloud used in the algorithm is not sampled, only the source point cloud is down-sampled.

**Figure 10:**
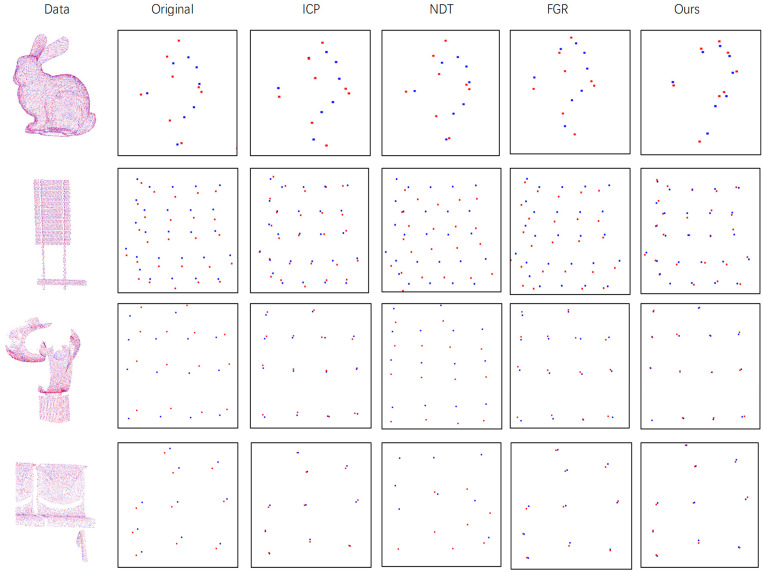
Performance on Gaussian Noise Data. The first column on the left of the figure is the original point cloud data, the second column is the detailed schematic diagram and the rest are the detailed diagrams after registration by each method.

**Gaussian Noise Data:** In order to more accurately simulate the presence of noise in the real point cloud and verify the ability of our algorithm to deal with noise, we added Gaussian noise to the experimental data. When preparing the simulation experiment, first perform a random rigid transformation on the original data, save the transformation results and then dither the transformed data within a certain range to achieve the purpose of Gaussian noise. The experimental results are shown in Table 3. Generally speaking, the ICP algorithm is the most accurate. The noise added in the experiment conforms to the normal distribution, so the expected value is 0, so the ICP effect is better, followed by our method, which is better than NDT and FGR. In terms of CD distance, because our method can improve the accuracy of the point cloud in detail, our method is the best overall. The qualitative results are shown in Figure 10. The red point cloud represents the transformed source point cloud, and the blue point cloud represents the target point cloud.

**Figure 11:**
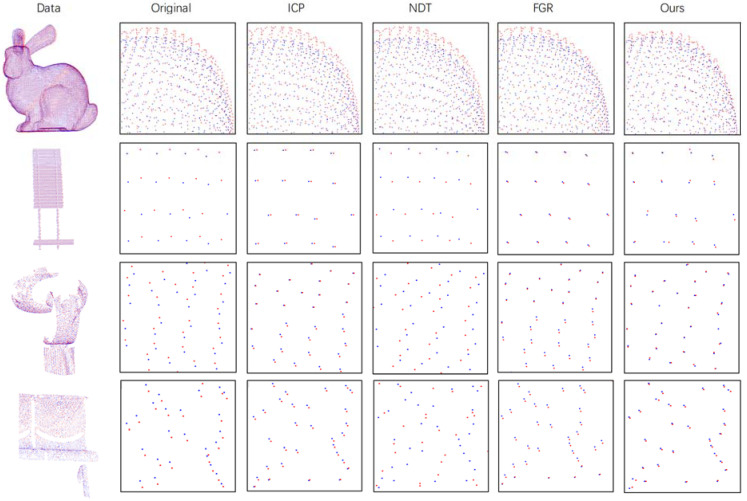
Performance on Distorted Data. The first column on the left of the figure is the original point cloud data, the second column is the detailed schematic diagram and the rest are the detailed diagrams after registration by each method.

**Distorted Data**: Due to the difference of each sensor, there may be a certain distortion between the point cloud and the point cloud. Therefore, a certain distortion is manually added to the experimental data. Since the point cloud is distorted, the true value of the transformation relationship cannot be obtained. Only applicable for CD distance evaluation. The experimental results are shown in Table 4. On the whole, our method is the best because it can adaptively correct the surrounding neighborhood of the point. The qualitative result is shown in Figure 11. The red point cloud represents the transformed source point cloud, and the blue Point cloud represents the target point cloud.

## 5. Conclusions

A method of virtual namesake point multi-source point cloud data fusion based on FPFH feature difference is proposed. It can synthesize the probability according to the F2 distance between the voxel center points and the existing points in the target point cloud. Then we generate virtual namesake points for registration according to the probability. The use of voxels, FPFH features and CNN estimation can improve the accuracy of point cloud fusion. We have compared our algorithm with the classic ICP algorithm, NDT algorithm and FGR algorithm. Through experiments and accuracy evaluation, in the case of clean point cloud and point cloud with different resolutions, our method has the same accuracy as the results of ICP and FGR algorithms and is better than the NDT algorithm. In the case of noise and distortion in the point cloud, our method is better than other algorithms. Since the FPFH feature is used in the calculation process, we will conduct a further study on the operating efficiency.

## Figures and Tables

**Table 1:** Performance on Clean Data.

Data	Method	Rotation Err. (°)	Translation Err. (m)	CD (m)
Bunny	ICP	0.002	0.00001	0.00000
	NDT	0.105	0.01832	0.00076
	FGR	0.001	0.00001	0.00000
	Ours	0.015	0.00321	0.00002
Sign Board	ICP	0.448	0.04120	0.00123
	NDT	0.228	0.08271	0.00227
	FGR	0.094	0.04397	0.00008
	Ours	0.195	0.05780	0.00025
Sculpture	ICP	0.001	0.00002	0.00000
	NDT	0.337	0.07969	0.00249
	FGR	0.017	0.00117	0.00000
	Ours	0.008	0.00204	0.00000
Chair	ICP	0.003	0.00000	0.00000
	NDT	2.373	0.09056	0.00232
	FGR	0.018	0.00128	0.00000
	Ours	0.022	0.00189	0.00000

**Table 2:** Performance on Different Resolution Data.

Data	Method	Rotation Err. (°)	Translation Err. (m)	CD (m)
Bunny	ICP	0.044	0.01323	0.00316
	NDT	0.201	0.01667	0.00366
	FGR	0.083	0.00559	0.00315
	Ours	0.032	0.00413	0.00310
Sign Board	ICP	0.111	0.02589	0.00141
	NDT	0.150	0.08755	0.00178
	FGR	0.414	0.27841	0.00321
	Ours	0.085	0.07789	0.00148
Sculpture	ICP	0.131	0.01742	0.00044
	NDT	0.448	0.06963	0.00228
	FGR	0.297	0.04777	0.00052
	Ours	0.201	0.01555	0.00046
Chair	ICP	0.027	0.00186	0.00002
	NDT	2.076	0.09368	0.00243
	FGR	0.201	0.01737	0.00243
	Ours	0.039	0.00260	0.00002

**Table 3:** Performance on Gaussian Noise Data.

Data	Method	Rotation Err. (°)	Translation Err. (m)	CD (m)
Bunny	ICP	0.004	0.00024	0.00126
	NDT	0.152	0.01740	0.00202
	FGR	0.080	0.00101	0.00132
	Ours	0.043	0.00810	0.00112
Sign Board	ICP	0.026	0.02363	0.00020
	NDT	0.211	0.09656	0.00197
	FGR	0.257	0.09921	0.00067
	Ours	0.115	0.08282	0.00018
Sculpture	ICP	0.016	0.00025	0.00001
	NDT	0.355	0.06781	0.00168
	FGR	0.197	0.01182	0.00002
	Ours	0.330	0.02407	0.00001
Chair	ICP	0.002	0.00003	0.00051
	NDT	2.442	0.09082	0.00227
	FGR	0.027	0.00197	0.00063
	Ours	0.012	0.00151	0.00055

**Table 4:** Performance on Distorted Data.

Data	Method	CD (m)
Bunny	ICP	0.00913
	NDT	0.01203
	FGR	0.01077
	Ours	0.00600
Sign Board	ICP	0.00007
	NDT	0.00169
	FGR	0.00010
	Ours	0.00018
Sculpture	ICP	0.00022
	NDT	0.00040
	FGR	0.00025
	Ours	0.00009
Chair	ICP	0.00015
	NDT	0.00166
	FGR	0.00016
	Ours	0.00010

## Data Availability

Not applicable.
